# Resting-state occipito-frontal alpha connectome is linked to differential word learning ability in adult learners

**DOI:** 10.3389/fnins.2022.953315

**Published:** 2022-09-15

**Authors:** Yan Huang, Yao Deng, Xiaoming Jiang, Yiyuan Chen, Tianxin Mao, Yong Xu, Caihong Jiang, Hengyi Rao

**Affiliations:** ^1^Center for Magnetic Resonance Imaging Research, Key Laboratory of Applied Brain and Cognitive Sciences, Shanghai International Studies University, Shanghai, China; ^2^School of Foreign Languages, East China University of Science and Technology, Shanghai, China; ^3^Institute of Linguistics, Shanghai International Studies University, Shanghai, China; ^4^Center for Functional Neuroimaging, Department of Neurology, University of Pennsylvania, Philadelphia, PA, United States

**Keywords:** resting-state brain connectome, EEG, alpha-band, individual differences, word learning ability

## Abstract

Adult language learners show distinct abilities in acquiring a new language, yet the underlying neural mechanisms remain elusive. Previous studies suggested that resting-state brain connectome may contribute to individual differences in learning ability. Here, we recorded electroencephalography (EEG) in a large cohort of 106 healthy young adults (50 males) and examined the associations between resting-state alpha band (8–12 Hz) connectome and individual learning ability during novel word learning, a key component of new language acquisition. Behavioral data revealed robust individual differences in the performance of the novel word learning task, which correlated with their performance in the language aptitude test. EEG data showed that individual resting-state alpha band coherence between occipital and frontal regions positively correlated with differential word learning performance (*p* = 0.001). The significant positive correlations between resting-state occipito-frontal alpha connectome and differential world learning ability were replicated in an independent cohort of 35 healthy adults. These findings support the key role of occipito-frontal network in novel word learning and suggest that resting-state EEG connectome may be a reliable marker for individual ability during new language learning.

## Introduction

More than one billion people are learning a foreign language each year. The ability to learn or speak a foreign language beyond one’s native tongue is particularly vital for professional and personal growth in the contemporary globalized world. While language learning is a fairly common endeavor in adulthood, it is generally not as successful as in childhood (Tagarelli et al., 2019). Foreign language acquisition, especially in adulthood, is characterized by considerable variability in the learning rate and ultimate attainment (Dörnyei and Skehan, 2003; Dörnyei, 2009). For some people, the process of learning a foreign language is time-consuming and challenging, while for others, high levels of language proficiency can be attained with relative ease and little time investment. Understanding the neural mechanisms responsible for such heterogeneity among late language learners is crucial for elucidating the nature of learning ability and further enhancing learning efficiency through precise neuromodulation.

From a network neuroscience perspective, the human brain is a complex network consisting of numerous functionally specialized brain regions and inter-regional connections (Hilger and Sporns, 2021). Analyzing resting-state functional connectome (rs-FC), the temporal synchronization of spontaneous neural activity between anatomically separated brain regions, is a powerful method to characterize neural network (Fox and Raichle, 2007; Smith et al., 2013; Vinals, 2016). Additionally, there is accumulating evidence that functional connectome profiles act as a unique “fingerprint” that can be used to predict inter-individual differences in behavior and cognition (Finn et al., 2015; Waller et al., 2017; Gratton et al., 2018; Lin et al., 2020). Given the pronounced variability in foreign language acquisition among adult learners, the rs-FC approach hold promise for investigating the neural basis of individual differences in language learning ability (Chai et al., 2016; Vinals, 2016). Recently, a growing number of neuroimaging studies have linked variability in resting-state brain connectome with specific language learning abilities, such as word learning ability (Veroude et al., 2010; Palomar-García et al., 2017; Schlaffke et al., 2017), sound learning ability (Ventura-Campos et al., 2013; Qi et al., 2019), and grammar learning ability (Antonenko et al., 2012). In general, these studies have provided compelling evidence that the strength of rs-FC within the language network can be predictive of individuals’ language learning ability. However, the majority of these research used functional magnetic resonance imaging (fMRI) to investigate the association between resting brain network and language learning behavior. Despite its relatively high spatial resolution, fMRI is an indirect measurement of neural activity through relatively slow hemodynamic changes with a temporal resolution in seconds (Hilger and Sporns, 2021). In contrast, functional connectome as measured by electroencephalography (EEG) has a much higher temporal resolution in milliseconds, therefore reflecting more real-time neural processes based on cross-region coupling of fast oscillations (Wirsich et al., 2020). As such, the oscillation-based EEG functional connectome is well-positioned to support long-range communication required for language learning processes that unfold on the timescale of milliseconds (Mostame and Sadaghiani, 2021; Sadaghiani et al., 2022).

A common method to quantitatively estimate the large-scale neuronal synchronization between cortical regions is the calculation of EEG coherence (Weiss and Mueller, 2003; Reiterer et al., 2005; Srinivasan et al., 2007). Coherence is a statistical measure of the average agreement in phase difference, weighted by amplitude, between two signals measured over time, and is frequency specific (Chorlian et al., 2009). High coherence between EEG signals recorded at two sites can be interpreted as intense functional interaction (degree of information flow) between the underlying neuronal networks (Weiss and Mueller, 2003). Albeit not many, studies into the relationship between resting-state electroencephalography (rsEEG) connectivity and language learning ability have, to date, yielded inconclusive findings. For instance, Prat et al. (2019) observed that increased alpha/beta-band coherences within and between right frontotemporal/posterior networks were associated with better second language (L2) learning outcome (e.g., L2 learning rate, ultimate declarative memory), whereas a recent study conducted by Kliesch et al. (2022) revealed that only the alpha-band connectivity between right-hemispheric fronto-temporal areas significantly correlated with the development of L2 complexity. The heterogeneous findings were probably caused by differences in sample age (i.e., younger and older groups), different measurements of language learning ability (i.e., L2 learning rate, memory, etc.), and methodological differences in EEG data collection and analysis.

In this study, we focused on one of the most essential components of language acquisition, namely novel word learning. The ability to rapidly learn new words in a variety of contexts is crucial to the development of a large vocabulary as well as literacy skills (Gaskell and Ellis, 2009; Lederberg and Spencer, 2009). However, unlike native language word acquisition among children, which often appears swift and effortless, the capability of learning novel words tends to be less efficient and highly variable among adults. In other words, adults vary in their ability to both learn and retain novel words, yet the origin of this inter-individual word learning variability remains elusive to date. Word learning ability is generally held as the capability to establish connections between novel words and their semantic referents (Kaushanskaya et al., 2013; Borgström et al., 2015, 2016; Hamza et al., 2020). In recent studies, different variants of (auditory or visual) paired-associate learning tasks, for example word-meaning association task (Krug et al., 2002; McDermott and Zerr, 2019) and word-picture/object association task (Peñaloza et al., 2016; Lany, 2018), have been adopted to measure word learning ability. To our knowledge, there was only one study that has examined the relationship between resting-state EEG connectivity and word learning effects in a small sample of *N* = 16 subjects (Nicolo et al., 2016). This study reported the positive correlation between alpha-band EEG coherence and learning performance when participants received active continuous theta burst stimulation before the learning task. However, it remains a challenge to replicate brain-behavioral associations from small sample and under-powered studies (Marek et al., 2022). In response, the goal of this study was to examine the relationships between the resting-state EEG connectome and differential word learning ability among a much larger sample of adult learners. EEG spontaneous activities in the alpha band (8–12 Hz) are of particular interest in this study since mounting evidence has supported the functional relevance of intrinsic alpha oscillations for the implementation of verbal learning and memory tasks (Williams et al., 2006; Klimesch, 2012; Brokaw et al., 2016; Mahjoory et al., 2019; Mkrtychian et al., 2021). As the dominant EEG signature of eyes-closed waking rest, alpha rhythm is one of the major EEG correlates of the default-mode network which covers a number of memory-related brain regions: including the hippocampus, parahippocampal cortex, and medial frontal cortex (Jann et al., 2009; Knyazev et al., 2011). Given the importance of memory in novel word learning, as well as the close relationship between resting-state alpha oscillations and verbal long-term memory (Brokaw et al., 2016; Mahjoory et al., 2019), we hypothesized that alpha-band EEG connectivity measured before tasks might also be correlated with differential word learning performance. Further, taking into account previous evidence on the functional role of the inferior fronto-occipital fascicle (IFOF) (i.e., the ventral pathway connecting the frontal cortex and the occipital cortex) in lexical-semantic processing (see Friederici and Gierhan, 2013 for a review), and evidence on the role of long-range connectivity between frontal and posterior brain regions in predicting memory-related performances (e.g., working memory, episodic memory) (Shaw et al., 2015; Fleck et al., 2016), we also expected that the strongest associations between rsEEG alpha coherence and word learning ability would be observed primarily in occipito-frontal network. In addition, while the majority of prior neuroimaging work on the neuroanatomical structures involved in word learning has primarily identified a left-lateralized network of regions, such as the left hippocampus, the left inferior frontal gyrus, and the left middle temporal gyrus (Breitenstein et al., 2005; Mestres-Missé et al., 2008; Shtyrov et al., 2010; Ye et al., 2011; López-Barroso et al., 2013), it has also been reported that both hemispheres may make important but different contributions in the process of acquiring new words (Borovsky et al., 2013; Riès et al., 2016). Therefore, we further aimed to uncover the hemispheric engagement in terms of the link between rsEEG connectome and word learning ability.

In summary, despite the growing interest in what determines individual differences in word learning ability, it is largely unknown whether they may in part depend on differences in spontaneous brain connectome patterns at rest. Here, we attempted to fill this gap by investigating whether and how pre-task brain connectome measured by EEG were related to the ability of acquiring novel words in adults. As indicated above, we expected that individual resting-state EEG alpha coherence between occipital and frontal regions could predict inter-individual variability in word learning ability.

## Materials and methods

### Participants

A total of 106 healthy, right-handed undergraduate students (50 males, mean age = 21.41 ± 2.20 years) participated in the study (see Table 1). They were recruited from several universities in China and received monetary compensation for their participation. All participants were native Chinese speakers who had received classroom-based English (L2) education for approximately 10 years (age of L2 acquisition = 8.23 ± 2.15). They had self-reported normal or corrected-to-normal vision and no history of neurological or language-related disorders. The study protocol was approved by the Ethics Committee of Shanghai International Studies University. All participants signed the informed consent prior to the experiment.

**TABLE 1:** Descriptive statistics for participants’ bio-data and behavioral task performance in two independent studies.

Participants’ bio-data					

	Total *N*	Age *M* (*SD*)	AoA*^a^ M* (*SD*)	Gender	
				
				Male	Female
Main study	106	21.41 (2.20)	8.23 (2.15)	50	56
Replication study	35	26.80 (7.84)	9.20 (2.75)	13	22

**Behavioral task performance**					

	**Measurements**	***Min***	***Max***	***M* (*SD*)**	***SE***

Main study	Novel-word learning task	6	60	39.97 (15.32)	1.49
	LLAMA B	10	100	56.93 (21.33)	2.07
Replication study	Novel-word learning task	9	60	43.37 (13.94)	2.36

*^a^*AoA, age of learning English under formal instruction.

### Materials

Learning materials comprised a list of 60 Pseudo-English-Chinese word pairs (see Table 1 in Supplementary Material). The number of word pairs to be learned was mainly determined by previous literature (e.g., Dahlström et al., 2011; Nakata, 2016; Batterink et al., 2017) and our pilot data suggested that 60 pseudo-English-Chinese word pairs were able to detect individual differences in word learning ability and avoid the ceiling or floor effects.

The pseudowords were all pronounceable and orthographically legal in English, varied in length from 6 to 8 letters with 2 syllables, and were controlled in terms of the orthographic neighborhood size measured by the Orthographic Levenshtein Distance (OLD20) (Yarkoni et al., 2008). OLD20 is computed as the mean Levenshtein Distance from a word to its 20 closest orthographic neighbors. The smaller the value of OLD20, the fewer steps (additions, deletions, or substitutions) required to convert a pseudoword into a real word. Given this, the OLD20 of all pseudowords in this study was kept at 3 or higher, with an aim to reduce the associative memory effect while retaining the English word-formation rules of these pseudowords. All pseudo-words used in this study were generated in accordance with the lexical features of real English words (e.g., phonology, orthography, word length, number of syllables) to improve the ecological validity.

Each English pseudoword (e.g., “plulfot”) was paired with a Chinese meaning (e.g., “草原”), and these Chinese equivalents were all two-character words selected from the *Modern Chinese Frequency Dictionary* (1986). Additionally, in order to control the difficulty and familiarity of these Chinese words, we calculated the number of strokes (*M* = 14.32, *SD* = 3.13) and word frequency (*M* = 0.0034, *SD* = 0.0027), and excluded the words with extreme values (≥ ± 2 *SD*). Similar to the literature (Lu et al., 2017), all Chinese words were concrete nouns that belonged to one of the following semantic categories: fruits, vegetables, animals, body parts, work, sports, clothing, furniture, electrical appliances, and natural scene.

### Procedure

**FIGURE 1:**
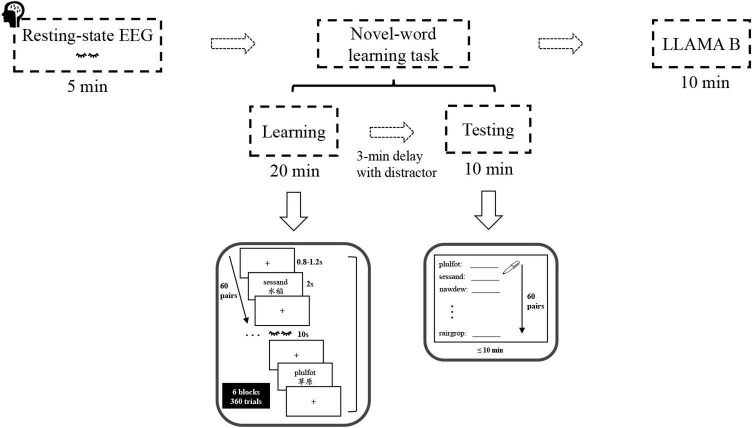
Experimental procedure and stimuli. The sequence of task sessions (top) and sample trials of the novel-word learning task (bottom).

Participants were asked to complete the language background questionnaire and a demographic survey prior to the eyes-closed resting-state EEG recording. Afterward, they completed the paired-associate novel-word learning task and the LLAMA B task in sequence (see Figure 1).

The novel-word learning task consisted of two stages: learning and testing. During learning, participants were instructed to learn and memorize the novel words and their Chinese meanings presented on the computer screen. Specifically, they studied 60 pairs of novel words (60 trials) over 6 rounds (6 blocks), with the pairs in each round presented only once in random order. In each block, word pairs were presented one at a time for 2 s each and were separated by a jittered inter-stimulus-interval (ISI: 0.8–1.2 s). A short break was granted after each learning block. Participants were then asked to perform a 3-min distractor task (psychomotor vigilance test), data of which were not analyzed in this paper. After that, participants were tested on their memory for the novel words using a cued-recall test (paper-and-pencil test). Specifically, they were required to write down the Chinese equivalent (e.g., “草原”) for each pseudo-English cue (e.g., “sessand”) within 10 min. For each participant, the test words (60 items) were displayed in a random order, and the number of words correctly recalled in the final cued-recall test was used as an index of word learning ability.

Finally, participants took the LLAMA B task, a subtest of the LLAMA language aptitude test battery. LLAMA B is essentially a vocabulary learning task, which measures the ability to learn a relatively large number of new words in a short space of time (Meara, 2005). At the stage of vocabulary learning, participants were allowed to learn word-picture pairs within 2 min, followed by a test phase where participants were asked to select one out of 20 picture stimuli when they were presented with a word. Notably, the vocabulary items to be learned were real words, derived from a northwest British Columbian indigenous language of Canada assumed to be unknown to most people. At the end of the LLAMA B task, the scores ranging from 0 to 100 were automatically calculated and displayed at the bottom of the screen. Overall, the entire experiment lasted for approximately 60 min per participant.

### Behavioral data analysis

In the novel-word learning task, the number of words correctly recalled in the final cued-recall test was calculated and used as an index of individual word learning ability for each subject for subsequent behavioral and EEG analysis. Correlation analysis was conducted between the novel-word learning performance and the LLAMA B scores to evaluate the validity of the novel-word learning task. The rank-based normal scores transformation (Blom’s method) (Solomon and Sawilowsky, 2009) was performed on the raw data of word learning scores to meet the assumption of normal distribution.

### Electroencephalography acquisition and preprocessing

Continuous EEG was recorded using a 32-channel BrainVision ActiCap active electrode system (BrainProducts GmbH, Germany) during resting conditions in which participants were required to stay awake, but with their eyes closed. Signals were bandpass filtered between 0.05 and 100 Hz and sampled at 500 Hz. Impedances for each channel were maintained below 5 KΩ before recording. The reference electrode was located at FCz and the ground electrode at AFz. During the eyes-closed rsEEG recording, participants were seated comfortably in a dimly lit, sound-proof experimental room. The rsEEG recording lasted for 5 min and was implemented before the behavioral tasks.

Offline EEG data preprocessing were performed using EEGLAB toolbox (version 14.1.1; Delorme and Makeig, 2004) and customized MATLAB (The MathWorks, Inc., Natick, United States) scripts. First, the continuous EEG data were re-referenced to the averaged mastoids, band-pass filtered between 1 and 40 Hz (50 Hz notched) with a linear finite impulse response (FIR) filter, and segmented into non-overlapping 2 s epochs. Subsequently, large artifacts (i.e., muscular artifacts, eye movements, eye blinks, etc.) were rejected manually by visual inspection. Further, the independent component analysis (ICA) and the automatic raw data inspection (max amplitude: ± 80 uV) were consecutively performed to identify and correct for the remaining artifacts. Finally, the artifact-corrected data were manually screened a second time to ensure data purity. On average, 148.26 (*SD* = 2.62) epochs per participant were selected for further analysis, accounting for 98.8% of the total epochs (150 epochs).

### Electroencephalography data analysis

**FIGURE 2:**
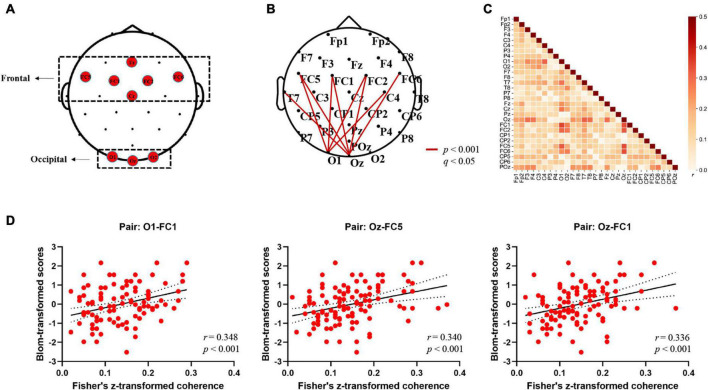
Electrodes in the regions of interest (ROIs) and alpha-band EEG coherence results (Fisher-*Z*). **(A)** Topographical grouping of electrodes, with red labels provided for the electrodes included in analyses. **(B)** Summarized topography of whole-brain (channel-to-channel) functional connectivity analysis in the alpha band (8–12 Hz). The thick red lines represent cortical connections where the connectivity strengths correlated with word learning performance significantly (FDR corrected: *p* < 0.001; *q* < 0.05). **(C)** Heat map of the correlations between channel-to-channel (29 × 29 pairs) coherence values in the alpha band (8–12 Hz) and word learning performance averaged across 106 participants. The color bar on the right represents the Pearson correlation coefficient (*r*). **(D)** Scatterplots depicting significant correlations between alpha-band coherence values and word learning performance at three exemplar electrode pairs (O1-FC1; Oz-FC5; Oz-FC1) (*p* < 0.001; *q* < 0.05).

The functional connectivity between distinct brain regions was estimated using EEG coherence (Petsche, 1997). First, considering the signal noise and the regions of interest, two mastoid electrodes (TP9 and TP10) and the electrode FCz were not retained for subsequent EEG coherence analysis (see Figure 2B for the remaining 29 sites). Then, the artifact-free data were transformed from the time domain into the frequency domain by using Fast Fourier Transform (FFT) (Cooley and Tukey, 1965). Finally, coherence between all possible electrode pairs [(29*28)/2 = 406 pairs] was calculated in the commonly studied frequency bands (delta: 1–4 Hz; theta: 4–8 Hz; alpha: 8–12 Hz; beta: 12–30 Hz; gamma: 30–40 Hz). Following previous literature (e.g., Hu and Zhang, 2018; Prat et al., 2019), coherence *C_*x*,y_* (*f*) between two channels *x* and *y* at a frequency *f* was computed according to the following formula:


$${\text{C}}_{x,y}\,\left(f\right)=\frac{{\text{P}}_{x,y}\,{\left(f\right)}^{2}}{{\text{P}}_{x}\,\left(f\right)\times {\text{P}}_{y}\,\left(f\right)}$$

Where the *P*_*x,y*_(*f*) is the cross-power density and the *P*_*x*_(*f*) and *P*_*y*_(*f*) are the power densities of *x* and *y*, respectively. The coherence value ranges from 0 to 1, with higher values representing stronger synchronization between two signals. The obtained coherence matrix for each participant was normalized using Fisher’s z-transformation. Based on the second hypothesis in this study, we sought to further elucidate the relationship between electrode/cluster-level coherence values and word learning ability. First, we performed the whole-brain analysis in which we extracted the coherence values of each participant between all electrode pairs (106 participant * 406 pairs * 5 frequency bands) and evaluated the correlation between EEG coherence for all possible pairwise combinations of electrodes (Fisher’s *Z*) and individual word learning performance (Blom-transformed scores). Further, to address the multiple comparison problem, the statistical *p*-value was corrected by False Discovery Rate (FDR) (Benjamini and Hochberg, 1995) method over the whole brain, and the values that passed the corrected threshold of *q* < 0.05 were highlighted in the following results part. Second, in order to further examine the long-range connections of an occipito-frontal network, we averaged the Fisher’s z-transformed coherence values between network pairs of particular interest in accordance with the topographical grouping of electrodes (Chai et al., 2019; see Figure 2A), and then estimated the Pearson correlation coefficient between the connectivity across two sub-clusters (Occipital region: O1, Oz, O2; Frontal region: Fz, FC1, FC2, FC5, FC6, Cz) and the Blom-transformed word learning scores. Moreover, one-way analysis of variance (ANOVA) with *post hoc* Bonferroni tests (*p* < 0.05) were performed to investigate whether there were significant differences in individual occipito-frontal coherence values across different learning ability groups. Specifically, all participants were divided into three learning ability groups based on the tertiles of word learning scores (Group High: scores ≥ 50; Group Intermediate: 33 < scores < 50; Group Low: scores ≤ 33). Third, hierarchical multiple regression analysis was further performed to evaluate the predictive effect of individual resting-state occipito-frontal alpha connectome (cluster-level) on word learning scores after controlling for demographic variables (age, gender). Subsequently, Pearson correlation coefficient (*r*) and root mean squared error (*RMSE*) between actual and predicted word learning scores were computed to evaluate the accuracy of prediction (Feng et al., 2018, 2019). Finally, the relationship between individual word learning performance and the left/right hemispheric occipito-frontal EEG connectivity was measured separately to compare the differences in the correlation coefficients between the two hemispheres (LH, Left hemisphere; RH, Right hemisphere).

### Validation study

We also conducted a validation study to determine whether the findings can be replicated in a different sample. An independent cohort of 35 healthy, right-handed adults (13 males, mean age = 26.80 ± 7.84 years; age range = 18–45) were recruited. All participants underwent 5 min eyes-closed rsEEG recording and then complected the same Pseudo-English-Chinese word learning task.

## Results

### Behavioral results

We observed robust inter-individual differences in the performance of the novel word learning task (see Table 1). On average, participants correctly recalled 39.97 words (range from 6 to 60, *SD* = 15.32) in the final cued-recall test. The LLAMA B scores also showed large inter-individual differences, with a mean score of 56.93 (*SD* = 21.33) and a range from 10 to 100. There was a significant positive correlation between the novel word learning task performance and the LLAMA B test scores [*r*_*s*_ (106) = 0.420, *p* < 10^–5^]. In other words, participants who performed better on the novel-word learning task also scored higher on the LLAMA B aptitude test. The results suggest that word learning ability is a relatively stable trait.

### Electroencephalography results

In order to reveal the relationship between resting-state EEG connectome and word learning ability, we first conducted the whole-brain analysis where the coherence values of all possible electrode pairs were computed and correlated with individual learning performance. Figure 2 presents a summary of the correlation results at the electrode level. First, as expected, significant positive correlations that had survived corrections for multiple comparisons (FDR) were found only in the alpha frequencies (8–12 Hz) over the whole scalp sites (*p* < 0.05, *q* < 0.05). Specifically, strong correlations were mainly observed between the alpha-band long-range electrode connections distributing over occipital [O1, Oz] and frontal regions [FC1, FC2, FC5, FC6] [*r* (106) = 0.309 ∼0.348, *p* < 0.001, *q* < 0.05] (see Figure 2B for details). On the other hand, no correlation reached significance at the corrected value of *q* < 0.05 in other frequency bands [Delta: *r* (106) = −0.149 ∼0.220, *p* > 0.02, *q* > 0.05; Theta: *r* (106) = −0.176 ∼0.271, *p* > 0.01, *q* > 0.05; Beta: *r* (106) = −0.187 ∼0.138, *p* > 0.05, *q* > 0.05; Gamma: *r* (106) = −0.165 ∼0.081, *p* > 0.05, *q* > 0.05], suggesting that the significant association between rsEEG connectome and word learning ability was specific to the alpha band. The matrix heat map in Figure 2C visualizes the whole-brain correlations between channel-to-channel coherence values in the alpha band and word learning outcome. Since no negative correlation reached significance at the uncorrected threshold of *p* < 0.05, the results of negative correlation (*r* < 0) were not displayed. In general, similar to the connectivity patterns in Figure 2B, the alpha-band coherence between occipital and frontal electrode pairs was more strongly correlated with word learning performance than the connectivity in other regions. For instance, the coherence values of O1-FC1, Oz-FC5, and Oz-FC1 pairs had the highest correlation with word learning performance among all electrode pairs (see Figure 2D).

**FIGURE 3:**
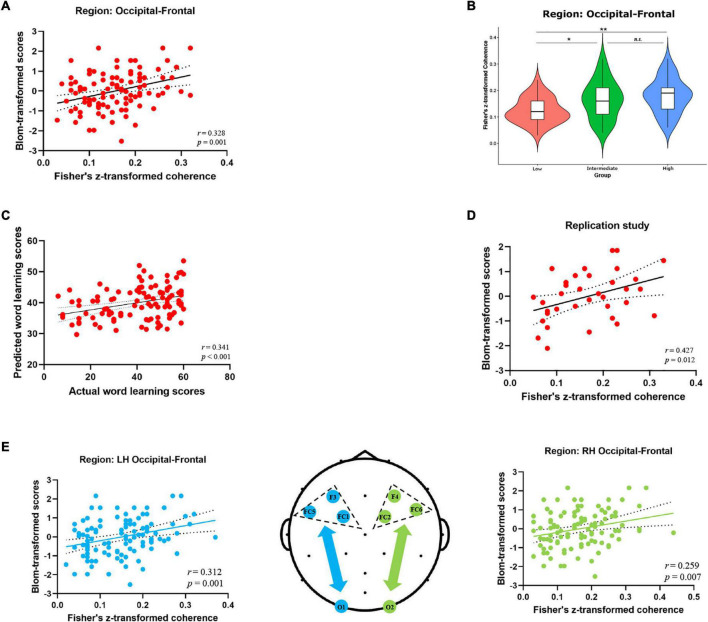
Alpha-band EEG coherence results (Fisher-Z) at the cluster level. **(A)** Correlation between the alpha-band coherence values over occipito-frontal regions (electrode sites: O1, Oz, O2, Fz, FC1, FC2, FC5, FC6, Cz) and word learning outcome. **(B)** Comparison of the occipito-frontal coherence values between three learning ability groups (High V.S. Intermediate V.S. Low) (*p < 0.05, **p < 0.01, Bonferroni corrected). **(C)** Correlation between actual and predicted word learning scores. **(D)** Correlation between occipito-frontal coherence and word learning outcome in the replication study (35 participants). **(E)** Correlation between the occipito-frontal coherence and word learning scores within the left hemisphere (blue scatterplot) and the right hemisphere (green scatterplot). The topographic map in the middle depicted the occipital and frontal electrodes of interest included in the cluster-level coherence analyses within the two hemispheres. Electrode sites in the left hemisphere were marked in blue, while those in the right hemisphere were marked in green.

Considering the relatively low spatial resolution of EEG, we also performed the regional EEG analysis by clustering neighboring electrodes in a priori defined ROIs (see Figure 2A) and examined the role of occipito-frontal network in novel word learning. First, as shown in Figure 3A, individual resting-state coherence between occipital and frontal regions significantly correlated with differential word learning performance [*r* (106) = 0.328, *p* = 0.001]. Likewise, the results of ANOVA showed significant differences in the occipito-frontal coherence between different learning ability groups [*F*(2, 103) = 7.585, *p* = 0.001, η2 p = 0.128] (see Figure 3B). It was revealed in the post hoc analysis (Bonferroni corrected) that the occipito-frontal region connectivity strength in the high-performance group (*M* = 0.178, *SD* = 0.065) was significantly stronger than that in the low-performance group (*M* = 0.124, *SD* = 0.048) (*p* = 0.001). Second, results of hierarchical regression analysis (see Table 2A) showed that (1) the change in *R*^2^ for alpha-band occipito-frontal coherence was significant (*F* = 12.951, *p* < 0.001) beyond the contribution of demographic variables (age, gender) and that (2) individual occipito-frontal coherence had a significant contribution (β = 0.335, *p* < 0.001) to the fit of the regression model. Furthermore, the model validity was evaluated by Pearson correlation coefficient (*r*) and *RMSE* between actual and predicted word learning scores. As shown in Figure 3C, there was a significant correlation between actual and predicted word learning scores [*r* (106) = 0.341, *p* < 0.001, *RMSE* = 14.611], suggesting that the regression model of rsEEG alpha-band connectivity had significant prediction power for word learning scores. Third, we performed the same correlation analysis within the two hemispheres, respectively. It turned out that the positive correlations between occipito-frontal coherence and word learning ability reached significance in both the left hemisphere [*r*_*LH*_ (106) = 0.312, *p* = 0.001] and the right hemisphere [*r*_*RH*_ (106) = 0.259, *p* = 0.007] (see Figure 3E).

**TABLE 2A:** Summary of hierarchical multiple regression results.

(A) Main study (106 participants)				

Predictor	β	*R* ^2^	Δ*R*^2^	*F*-change
Step 1		0.004	0.004	0.197
Age	0.003			
Gender	0.062			
Step 2		0.116	0.112	12.951***
Age	–0.010			
Gender	0.056			
Occipito-frontal coherence	0.335***			

Last but not least, the main findings of EEG regional analysis were replicated in the independent validation study. Specifically, individual resting-state alpha-band coherence averaged between occipital and frontal regions significantly correlated with word learning performance [*r* (35) = 0.427, *p* = 0.012] (see Figure 3D). Moreover, the pre-task occipito-frontal coherence in the alpha band could significantly predict final word learning scores (β = 0.466, *p* = 0.006) beyond the contribution of demographic variables (age, gender) (see Table 2B).

**TABLE 2B:** 

(B) Replication study (35 participants)				

Predictor	β	*R* ^2^	Δ*R*^2^	*F*-change
Step 1		0.093	0.093	1.586
Age	–0.303			
Gender	–0.012			
Step 2		0.300	0.207	8.875**
Age	−0.314^+^			
Gender	–0.110			
Occipito-frontal coherence	0.466**			

^+^0.05 < p < 0.06, **p < 0.01, ***p < 0.001; Gender: 1 = male, 2 = female.

## Discussion

With a focus on a network perspective to analyze the underlying neural correlates of word learning ability, the present study examined the associations between resting-state brain connectome computed from EEG coherence and individual word learning ability in a large cohort of 106 participants. Results showed that (1) higher alpha-band functional connectivity between occipital and frontal area was associated with better word learning performance, and that (2) the rs-FC within the occipito-frontal network could predict word learning scores significantly beyond the contribution of age and gender. In addition, the significant positive correlations between resting-state occipito-frontal alpha connectome and differential world learning ability were replicated in an independent cohort of 35 healthy adults. Taken together, these results have provided consistent evidence that intrinsic brain connectome, as measured by resting-state EEG, may be a physiological marker to predict differential ability in novel word learning. To the best of our knowledge, this study takes a pioneering step forward in establishing the link between resting-state EEG alpha connectome and individual word learning ability in adult learners, which provides novel insights into the neural mechanisms underlying differences in word learning ability.

Behavioral data revealed robust individual differences in novel word learning performance, which well correlated with the participants’ scores in the language aptitude test. As mentioned above, the LLAMA B test is a well-established and effective paradigm to measure individual word learning ability (Meara, 2005). However, it is different from the novel-word learning task in terms of learning methods (word-meaning pairings vs. word-picture pairings), learning content (pseudowords vs. real words), and learning difficulty (60 vocabulary items vs. 20 vocabulary items). Therefore, based on our findings, we may tentatively infer that (1) the novel-word learning task is of relatively high validity in measuring individual word learning ability and that (2) word learning ability might be a stable trait, since the word learning performance was highly correlated across tasks administered with different learning materials.

The idea of correlating resting-state brain connectome with language learning ability is hardly new (Chai et al., 2016; Vinals, 2016; Palomar-García et al., 2017; Schlaffke et al., 2017). Most of these attempts, however, rely on fMRI recordings and thus suffer from the methodological limitations and biases. According to a recent study (Marek et al., 2022), the primary challenge in the fMRI research has been replicating these brain-behavioral associations, since most of these studies have typically relied on a small sample size (the median neuroimaging study sample size is about 25), resulting in statistically under-powered studies, inflated effect sizes and replication failures. This study used EEG and aimed to examine whether robust individual differences in acquiring novel word meanings in adult learners can be traced back to pre-task brain oscillatory mechanisms and whether the findings could be reproducible in independent cohorts. As expected, we observed positive correlations between resting-state EEG alpha coherence between occipital and frontal regions and individual word learning performance across two independent samples. First of all, this result has added to previous evidence for the central role of alpha-band oscillations on word learning and memory (Klimesch, 1999, 2012; Williams et al., 2006; Brokaw et al., 2016; Mahjoory et al., 2019; Mkrtychian et al., 2021). Alpha rhythm has been reported as the primary EEG correlate of the fMRI-defined default mode network, which comprises a number of memory-related brain regions, including the hippocampus, para-hippocampal cortex, and medial frontal cortex (Jann et al., 2009; Knyazev et al., 2011; Brokaw et al., 2016). Since the learning ability of novel words was mainly assessed by the number of words (meanings) correctly recalled in the delayed cued-recall test, the word learning performance in this study was largely dependent on episodic memory. As such, the neural oscillatory patterns in the alpha frequency band might be functionally linked to word learning ability, at least in the initial stage of word acquisition. Secondly, from the perspective of functional connectome, our results suggest that alpha-band EEG coherence between the occipital and frontal regions could be a potential neural marker for word learning ability. Findings from this study not only corroborated the predicting effect of rsEEG connectivity on individual language learning abilities (Fleck et al., 2016; Prat et al., 2019; Kliesch et al., 2022), but also extended prior findings that the association between alpha-band connectivity strength and word learning performance held even in the absence of neuromodulatory interventions (Nicolo et al., 2016). Further, it is essential to consider the reasons why rsEEG connectivity between occipital and frontal regions is positively correlated with word learning performance since few studies have directly reported this association so far. Indeed, it has been shown that the language network identified by resting functional connectivity exhibits highly reproducible patterns consistent with those reported in task-based brain imaging studies (Tomasi and Volkow, 2012). Previous fMRI studies have indicated that when processing word meanings there is an increased activation not only in high-level “semantic hubs,” such as the inferior frontal cortex (Devlin et al., 2003), the anterior temporal cortex (Mion et al., 2010), or the inferior parietal cortex (Bonner et al., 2013), but also in early visual regions of the ventral visual stream (Pulvermüller, 2013). Further, a recent study conducted by Borghesani et al. (2016) has confirmed the “gradient semantic encoding” hypothesis that visuo-perceptual aspects of written words (such as real word size) appear to be encoded primarily in posterior occipital regions, while conceptual aspects (such as semantic category) appear encoded primarily in anterior temporal areas. In this case, the functional connectivity between the visual regions (occipital cortex) and the “sematic hubs” (across frontal and temporal cortex) might indicate the integrated encoding of different dimensions (perceptual and conceptual aspects) of word meaning. On the other hand, accumulating neuroimaging studies have consistently identified the crucial involvement of the left ventral occipitotemporal cortex (Ludersdorfer et al., 2019; Dong et al., 2021) as well as the left inferior frontal cortex (Gough et al., 2005; Mestres-Missé et al., 2008) in visual word processing. Specifically, the left ventral occipittemporal cortex is functionally specialized to orthographic processing (Cohen et al., 2000) and its middle part has even been labeled as the visual word form area (VWFA). Additionally, the left inferior frontal region provides the substrate for integrating parallel linguistic streams, including semantic and phonological information (Fiez, 1997; Gough et al., 2005). In this study, functional connectivity between visual orthographic processing and core semantic processing region has been associated with word learning abilities, suggesting that the coordination of brain regions supporting visual and semantic processing is crucial for successful acquisition of novel words (i.e., form-meaning associative learning). Another remarkable finding was the similar correlation patterns observed in the two hemispheres (see Figure 3E). In other words, the resting brain networks involved in word learning were more widely distributed and there was no hemispheric difference in predictive effects. Indeed, our data are consistent with a recent study showing that form-meaning associative learning elicited activations in a wide neural network including regions required for word processing (i.e., the bilateral inferior frontal gyrus and the occipitotemporal cortex) (Qu et al., 2021). Acquired brain lesion research also point to the importance of the integrity of both the LH and RH in early vocabulary development (Thal et al., 1991; Eisele and Aram, 1993). Generally, drawing on previous neuroimaging evidence as well as our research findings, we tentatively conclude that alpha-band connectivity strength of the bilateral occipito-frontal network measured by EEG at rest is coupled with one’s word learning abilities. This finding is of great significance in revealing the possible neural indicator of word learning ability from the perspective of EEG-derived functional connectome.

Meanwhile, several limitations of the present study should be noted. First, we only used pseudo-English words as learning materials and it remains unclear whether these findings can be generalized to real word learning. Future study may use authentic learning materials, such as rare words in less popular languages, to replicate our findings in real-word language learning scenarios. Second, we only used the number of words correctly recalled in the paired-associate novel-word learning task as an index of word learning ability. Future studies may use other indexes, such as the speed or the duration of learning, to replicate our findings. Third, word acquisition is a complex process involving multiple cognitive functions, such as attention and memory which cannot be studied by resting-state EEG. Further studies are needed to use carefully designed learning paradigms to distinguish specific cognitive processes and the underlying event-related potential (ERP) components during novel word learning. Fourthly, while oscillation-based functional connectivity has provided a unique insight into the underlying neural mechanisms of distinct abilities in acquiring novel words, the limited spatial resolution of EEG has also restrained us from making conclusions about which brain regions are involved in the network. This intrinsic methodological limitation could possibly be overcome by employing the simultaneous EEG-fMRI technique, which might improve both temporal and spatial resolution. Finally, the cross-sectional design of this research has limited us to explore the underlying causal relationships between rsEEG alpha connectome and word learning ability. Further studies using non-invasive neuromodulation technique are needed to determine whether the increased alpha-band connectivity could enhance the word learning performance.

## Conclusion

Using a pseudo-word leaning task in two independent studies, we demonstrated that pre-task resting-state alpha-band coherence between occipital and frontal regions measured by EEG predicted differential word learning performance in adult learners. These results provide consistent evidence supporting the key role of occipito-frontal network in novel word learning and suggest that resting-state EEG connectome may be a reliable marker for individual learning ability during new language acquisition. The findings may have important implications for the diagnosis and intervention of individuals with word learning deficits. More researches are needed to replicate and generalize our findings in younger learners during real-word new language acquisition.

## Data availability statement

The data that support the findings of this study are available from the corresponding author upon reasonable request with the need for a formal data sharing agreement and project outline.

## Ethics statement

The studies involving human participants were reviewed and approved by the Ethics Committee of Shanghai International Studies University. The patients/participants provided their written informed consent to participate in this study.

## Author contributions

HR and YH conceived the project and wrote the manuscript. YH conducted the study and collected and analyzed the data with the help of YD, YC, XJ, CJ, and TM. All authors reviewed the manuscript and approved the final version of the manuscript for submission.
