# Supplementary material for: Resting-state occipito-frontal alpha connectome is linked to differential word learning ability in adult learners
Source: Front Neurosci. 2022 Sep 15;16:953315. doi: 10.3389/fnins.2022.953315 (PMC9521374; doi:10.3389/fnins.2022.953315)
Supplement: Supplementary file 1 [file Table_1.DOCX]

Supplementary Material

## Supplementary Table 1. 60 (Pseudo)English-Chinese word pairs

| **Pseudoword** | **Meaning** | **Pseudoword** | **Meaning** | **Pseudoword** | **Meaning** |
| --- | --- | --- | --- | --- | --- |
| plulfot | 草原(grassland) | foctoun | 厨师(cook) | visture | 大脑(brain) |
| nawdew | 地板(floor) | jimpsen | 大海(sea) | mueboun | 厕所(washroom) |
| bettreet | 电脑(computer) | bodmig | 黄瓜(cucumber) | muckheam | 电灯(lamp) |
| fludpet | 耳朵(ear) | lussan | 会计(accountant) | sudnage | 公园(park) |
| kursten | 画家(painter) | rephern | 肌肉(muscle) | tettuke | 空调(air conditioner) |
| elpeme | 骆驼(camel) | comfave | 金鱼(goldfish) | osgion | 花生(peanut) |
| chefawn | 帽子(hat) | mossack | 老板(boss) | sostack | 蚂蚁(ant) |
| rairgrop | 眉毛(eyebrow) | stospelt | 轮船(ship) | purwase | 棉花(cotton) |
| rartcay | 泥土(dirt) | lifuch | 律师(lawyer) | lylien | 森林(forest) |
| provuth | 农民(farmer) | pivrol | 芒果(mango) | shatub | 士兵(soldier) |
| cirweat | 苹果(apple) | gonipt | 皮肤(skin) | pirehin | 司机(driver) |
| dertgoll | 青蛙(frog) | clactean | 沙发(sofa) | recuck | 头发(hair) |
| fatause | 舌头(tongue) | soltoor | 商店(store) | jenoor | 土豆(potato) |
| sessand | 书店(bookstore) | conswist | 树叶(leaf) | mattlic | 乌龟(tortoise) |
| yappal | 太阳(sun) | fiqutz | 衬衫(shirt) | soxpien | 学校(school) |
| jukemop | 西瓜(watermelon) | mectoun | 椅子(chair) | rezell | 血液(blood) |
| pregild | 相机(camera) | copfome | 山峰(mountain) | terfey | 牙齿(teeth) |
| spactien | 小麦(wheat) | pirrar | 月亮(moon) | modlaft | 医院(hospital) |
| preabon | 心脏(heart) | caggack | 足球(football) | vexuup | 玉米(corn) |
| tixlet | 银行(bank) | zopock | 鼻子(nose) | coggon | 阳台(balcony) |
